# *TMPRSS6* Non-Coding Variants in the Expression of Iron Refractory Iron Deficiency Anemia in Monoallelic Subjects

**DOI:** 10.3390/genes17010074

**Published:** 2026-01-08

**Authors:** Vera Hoving, Albertine E. Donker, Roel J. P. Smeets, Bert P. W. J. van den Heuvel, Saskia E. M. Schols, Dorine W. Swinkels

**Affiliations:** 1 Department of Hematology, Radboud University Medical Center, Geert Grooteplein Zuid 10, 6525 GA Nijmegen, The Netherlands; 2 Radboud Iron Expertise Center, Radboud University Medical Center, Geert Grooteplein Zuid 10, 6525 GA Nijmegen, The Netherlands; 3 Department of Pediatrics, Máxima Medical Center, De Run 4600, 5504 DB Veldhoven, The Netherlands; 4 Translational Metabolic Laboratory, Department of Human Genetics, Radboud University Medical Center, Geert Grooteplein Zuid 10, 6525 GA Nijmegen, The Netherlands; 5 Radboud Laboratory for Diagnostics, Department of Laboratory Medicine, Radboud University Medical Center, Geert Grooteplein Zuid 10, 6525 GA Nijmegen, The Netherlands; 6 Sanquin Blood Bank, Sanquin Diagnostics BV, Plesmanlaan 125, 1066 CX Amsterdam, The Netherlands

**Keywords:** IRIDA, *TMPRSS6*, non-coding variants, monoallelic

## Abstract

Background: Iron-refractory iron deficiency anemia (IRIDA) is a rare hereditary disorder caused by pathogenic variants in *TMPRSS6*, characterized by microcytic anemia, low circulating iron levels, and inappropriately high hepcidin levels. Although IRIDA is typically an autosomal recessive disorder, some individuals with a monoallelic pathogenic exonic *TMPRSS6* variant exhibit the phenotype, suggesting additional contributing factors. The mechanisms underlying monoallelic IRIDA remain unclear, complicating diagnosis. This study aimed to investigate the potential role of non-coding *TMPRSS6* variants and polygenic inheritance in monoallelic IRIDA. Methods: We performed full-gene sequencing of *TMPRSS6* in a cohort of 27 subjects, including 6 families (7 symptomatic monoallelic, 7 asymptomatic monoallelic, and 4 wild-type subjects) and 9 isolated symptomatic monoallelic subjects. Whole-exome sequencing of other iron-regulating genes was conducted to evaluate polygenic inheritance. Non-coding variants were assessed for inheritance patterns using family segregation analysis, when available, and for pathogenic potential using in silico prediction tools. Results: Sequencing identified 219 non-coding variants, of which 31 (14 *trans-*inherited and 17 with unknown inheritance) were exclusive to symptomatic subjects. Two *trans-*inherited variants (rs80140288 (c.229+945C>T) and rs146953827 (c.230-938_230-937del)) were predicted to affect splicing, while two additional variants (rs78987624 (c.-7001G>A) and rs117575523 (c.*503C>G)) were located in regulatory regions (with unknown inheritance). Whole-exome sequencing did not support polygenic involving other iron-regulating genes. Conclusions: This study highlights four candidate non-coding variants that may contribute to IRIDA expression in monoallelic subjects, offering new insights into its genetic basis. Functional validation is required to confirm their role in disease pathogenesis, refine genotype-phenotype correlations, and improve diagnostic accuracy in monoallelic IRIDA.

## 1. Introduction

Iron-refractory iron deficiency anemia (IRIDA) is a rare hereditary disorder caused by pathogenic variants in the *TMPRSS6* gene, encoding matriptase-2, a key regulator of hepatic hepcidin synthesis [1,2,3]. Dysfunctional matriptase-2 leads to excessive hepcidin production, which impairs intestinal iron absorption and iron mobilization from macrophages, resulting in iron-restricted erythropoiesis with resistance to oral iron therapy [4].

Clinically, IRIDA manifests as an iron distribution disorder, with low iron availability despite sufficient iron stores, progressing to absolute iron deficiency over time. Hallmark laboratory findings include microcytic anemia, low circulating iron (reflected by serum iron and transferrin saturation (TSAT)), and inappropriately high hepcidin relative to circulating iron levels [3,5]. While IRIDA is typically inherited in an autosomal recessive pattern, we and others have reported subjects who express the IRIDA phenotype with only a single pathogenic exonic *TMPRSS6* variant [6,7,8,9]. The mechanism underlying this monoallelic expression of IRIDA remains unclear, complicating diagnosis and clinical management.

In our previous work, we explored several hypotheses to explain this phenotypic variability in monoallelic IRIDA, including the potential contribution of epigenetic regulation, environmental modifiers, and additional genetic factors [6]. In line with these hypotheses, the present study investigates the role of non-coding *TMPRSS6* variants in IRIDA expression in monoallelic subjects by performing full-gene sequencing of *TMPRSS6*. Secondary objectives include evaluating the co-inheritance and prevalence of these variants. To exclude polygenic inheritance, whole-exome sequencing (WES) of other iron-regulating genes was conducted. By identifying additional genetic contributors, this study aims to clarify the pathophysiology of monoallelic IRIDA and improve diagnostic accuracy for affected individuals.

## 2. Materials and Methods

### 2.1. Study Population

This monocenter study used samples from monoallelic IRIDA subjects who had previously provided written informed consent for inclusion in the Radboud Iron Biobank. Subjects were identified at our specialized tertiary referral center for disorders of iron metabolism. Samples were collected during routine outpatient clinical care, alongside standard diagnostic procedures, avoiding additional invasive testing.

The study population comprised symptomatic monoallelic IRIDA subjects and, when available, their relatives, including asymptomatic monoallelic subjects and wild-type *TMPRSS6* subjects identified through family screening (Box 1).

Box 1 **Definitions. Bold is used to clarify the different definitions.**

**Symptomatic monoallelic IRIDA subjects**
Individuals carrying a single exonic *TMPRSS6* variant (pathogenicity class ≥ 3) who express the IRIDA phenotype [1].

**Asymptomatic monoallelic IRIDA subjects**
Relatives who share the same exonic *TMPRSS6* variant as their symptomatic family member but do not express the IRIDA phenotype.

**Wild-type *TMPRSS6* subjects**
Relatives of symptomatic monoallelic subjects who lack an exonic *TMPRSS6* variant and do not express the IRIDA phenotype.

The IRIDA phenotype was defined by microcytic anemia, low circulating iron (serum iron and TSAT < 10%, or <15% with iron supplementation) in the absence of inflammation, and partial or no response to oral iron therapy [6]. Other causes of iron-refractory anemia (e.g., autoimmune gastritis, celiac disease, *Helicobacter pylori*, and other gastrointestinal infections) were excluded when applicable. The TSAT/hepcidin ratio was used to support IRIDA phenotype confirmation (in bi-allelic IRIDA, TSAT/hepcidin ≤ 2.5th percentile relative to age indicated diagnosis) [3,5,10].

The monoallelic IRIDA genotype was determined by the presence of a single exonic *TMPRSS6* variant, classified as clearly pathogenic (class 5), likely to be pathogenic (class 4) or uncertain significance (class 3) based on joint English and Dutch practice guidelines [11,12].

This study received approval from the Medical Research Ethics Committee (MREC) Oost-Nederland (protocol number 2017-3270).

### 2.2. Amplification and Sequencing of TMPRSS6

DNA samples were amplified using long-range polymerase chain reaction (LR-PCR) to obtain sufficient *TMPRSS6* DNA for sequencing (Supplemental Information S1). Amplification was performed in six fragments, and PCR products were analyzed by gel electrophoresis. Sequencing was carried out using SMRT PacBio technology.

### 2.3. Data Analysis and Variant Classification

Variants were categorized into five classes based on available evidence, and allele frequencies were assessed using the Genome Aggregation Database. In silico tools, although not yet fully validated for non-coding variants, were applied to evaluate genomic location (e.g., regulatory regions), splicing effects using computational tools (Alamut Visual Plus 1.4), and predicted pathogenicity based on available classification frameworks [13]. Transcript *NM_153609.3* served as the reference sequence.

### 2.4. Identifying Candidate Non-Coding Variants

**Figure 1:**
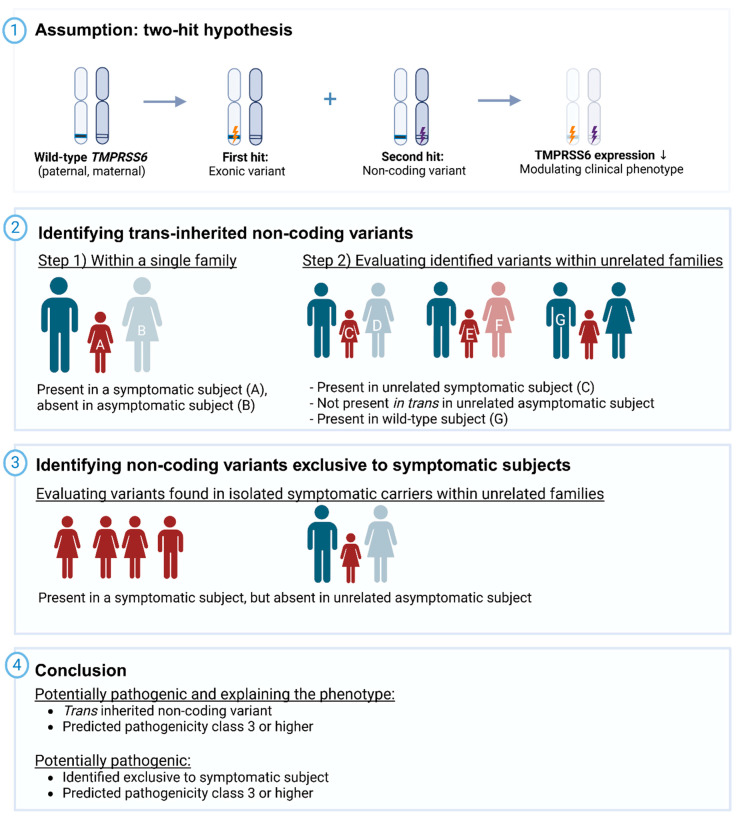
Stepwise approach for identifying candidate non-coding variants contributing to the IRIDA phenotype. (Panel 1) We assumed a two-hit theory in which a previously diagnosed exonic *TMPRSS6* variant (pathogenicity class ≥ 3) acts as the main risk factor for IRIDA. A non-coding variant on the opposite allele (*in trans*) may further contribute to IRIDA expression in symptomatic monoallelic subjects. Like exonic variants, these non-coding variants are expected to cause *TMPRSS6* loss-of-function. Variants were considered potentially pathogenic and explanatory if *trans-*inherited with the exonic variant and classified as pathogenicity class ≥ 3. Cis-inherited variants were considered unlikely to contribute to IRIDA expression. (Panel 2) We first identified *trans-*inherited non-coding variants within a single family. A non-coding variant was considered *trans-*inherited if present in a symptomatic subject (A) but absent in their asymptomatic relative (B). We then evaluated its presence across unrelated families to determine its potential contribution to phenotype expression: Potentially pathogenic/explanatory: present in another symptomatic subject (C) but absent in their related asymptomatic subject (D). Non-pathogenic: absent in a symptomatic subject of another family (E) but present in their asymptomatic subject (F); considered *trans-*inherited in that asymptomatic subject (F) and therefore deemed non-pathogenic, not explaining the phenotype in subject A. Cis-inherited: present in both a symptomatic (C) and asymptomatic (D) subject; unlikely to explain that family’s phenotype but could still contribute in symptomatic subject A. Uncertain significance: present in a wild-type subject (G) lacking the primary exonic variant. (Panel 3) For symptomatic subjects without included relatives, we prioritized non-coding variants found exclusively in symptomatic subjects. To confirm exclusivity, we examined these variants across unrelated families: Unlikely pathogenic: variant also present (*in trans*) in an asymptomatic subject. Possible contributor: variant present in wild-type subjects, where its clinical significance remains uncertain. ↓ = reduced.

We assumed a two-hit theory, in which the previously diagnosed (likely) pathogenic exonic *TMPRSS6* variant acts as the primary risk factor for IRIDA, while a non-coding variant in trans could contribute to IRIDA expression in symptomatic monoallelic subjects (Figure 1). Similar to exonic variants, these non-coding variants were expected to result in *TMPRSS6* loss-of-function [1]. Variants were prioritized as potentially pathogenic if *trans-*inherited with the exonic variant and classified as pathogenicity class ≥ 3.

For symptomatic subjects with available relatives, *trans-*inherited non-coding variants were identified through segregation and linkage disequilibrium analyses. For isolated symptomatic subjects (e.g., those without family data), non-coding variants unique to these individuals were prioritized (Figure 1).

### 2.5. Whole-Exome Sequencing

WES of additional iron-regulating genes was performed in all symptomatic subjects and asymptomatic relatives to exclude polygenic inheritance as a factor in phenotypic variation [14,15]. Iron-regulating genes were defined as those directly involved in cellular or systemic iron metabolism. Gene selection was based on their OMIM descriptions, excluding those associated with severe or unrelated conditions to minimize unsolicited findings, given the retrospective nature of this study (Supplementary Table S1).

Exome sequencing was conducted using an Illumina HiSeq2000TM at BGI-Europe following exome enrichment with the Agilent SureSelectXT Human All Exon 50 Mb Kit. Reads were aligned using Burrows-Wheeler Alignment (BWA), variants were called with Genome Analysis Toolkit (GATK), and annotation was performed by the Genetics Department of the Radboudumc using an in-house developed pipeline. All reported variants were confirmed through independent testing, including Sanger sequencing.

## 3. Results

**Figure 2:**
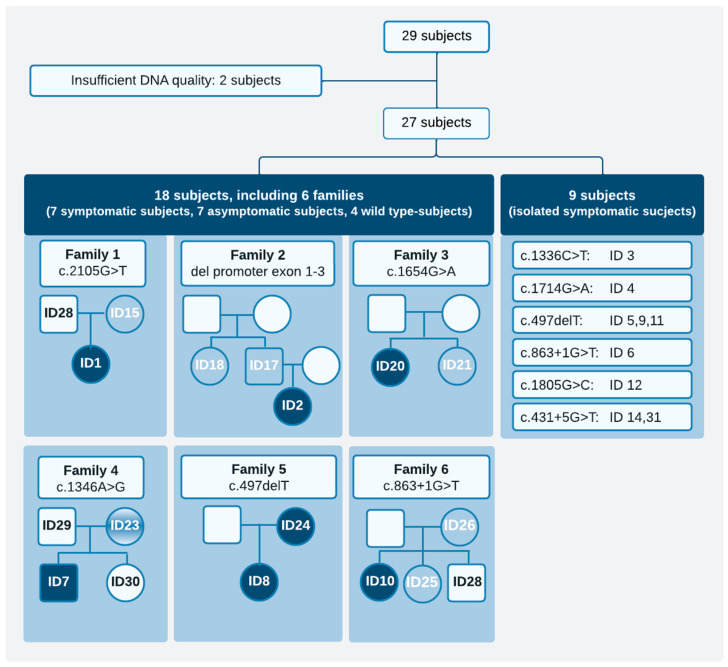
Overview of the study population. Monoallelic exonic *TMPRSS6* variants (pathogenicity class ≥ 3) are shown. (**Left**): Pedigrees of six included families. (**Right**): isolated symptomatic subjects, grouped by exonic *TMPRSS6* variant. Color legend: Dark blue—symptomatic; medium blue—asymptomatic subjects; light blue—wild-type *TMPRSS6*. Empty symbols indicate subjects with no additional data. Symbols: □ male, ○ female.

Our study population consisted of 27 subjects: 9 symptomatic subjects without family data (isolated symptomatic subjects) and 6 families, comprising 7 symptomatic subjects, 7 asymptomatic subjects, and 4 wild-type subjects (Figure 2, Supplementary Table S2). One family (Family 5) included two symptomatic monoallelic subjects (mother and daughter; Figure 2).

Demographic and laboratory characteristics of the population are summarized in Supplementary Table S2 and described in detail by Hoving et al. [6].

### 3.1. Identification of Non-Coding Variants Potentially Modulating IRIDA Phenotype

**Figure 3:**
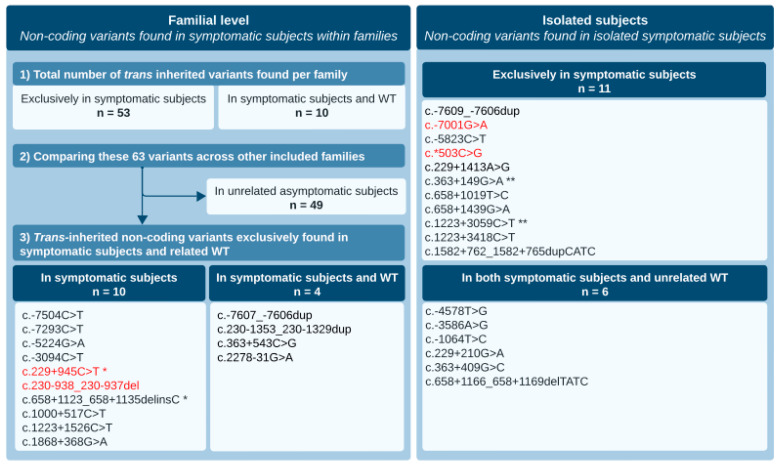
Overview of non-coding variants exclusive to symptomatic subjects. (**left**) The column outlines variants identified through familial segregation analysis, resulting in 14 *trans-*inherited non-coding variants: 10 present only in symptomatic subjects and 4 found in both symptomatic subjects and WT relatives. (**right**) The column shows 17 non-coding variants identified in isolated symptomatic subjects: 11 exclusive to symptomatic subjects and 6 present in both symptomatic subjects and unrelated WT-subjects. Variants predicted to be potentially pathogenic by in silico analysis are marked in red. * Variants also found in isolated symptomatic subjects. ** Variants also found in both symptomatic subjects of Family 5, considered in linkage disequilibrium due to the presence of the pathogenic exonic c.497delT in all carriers of this non-coding variant. Abbreviations: WT: wild-type.

Full-sequencing of *TMPRSS6* revealed 245 variants, with 219 in non-coding regions. Of these, 202 were identified within families. After excluding cis-inherited variants, 63 *trans-*inherited non-coding variants remained and were considered potentially contributing to the phenotype (Figure 3, left column). Cross-family analysis further revealed that 49 of these variants were also present in trans in unrelated asymptomatic subjects, leaving 14 *trans-*inherited variants: 10 exclusive to symptomatic subjects and 4 also found in wild-type subjects (Table 1; Figure 3, left column).

Analysis of the 9 isolated symptomatic subjects identified 17 additional non-coding variants: 11 exclusive to isolated subjects and 6 shared with unrelated wild-type subjects (Table 2; Figure 3, right column). Notably, 2 of these 11 exclusive non-coding variants (c.363+149G>A and c.1223+3059C>T) were observed in both symptomatic monoallelic subjects of Family 5.

### 3.2. Classification of Non-Coding Variants Potentially Linked to Phenotype Expression

In total, 31 non-coding variants were found exclusively in symptomatic subjects (Figure 3). The majority (24 out of 31) involved single-nucleotide changes. Clinical significance was assessed based on in silico predicted pathogenicity, inheritance mode, and prevalence.

Four were classified as class 3 variants (variants of unknown significance, VUS), potentially contributing to IRIDA (Table 3. Two were located in intron 2, *trans-*inherited and predicted to affect splicing:

- rs80140288 (c.229+945C>T): Identified exclusively in two unrelated symptomatic subjects (ID 9, ID 24) carrying the exonic c.497delT variant. This variant has a low minor allele frequency (MAF, 0.53%). Its absence in ID 8 from Family 5 (which includes ID 8 and ID 24) suggests *trans-*inheritance within this family. If proven pathogenic, this variant could indicate different underlying causes for the phenotype in the two symptomatic subjects.
- rs146953827 (c.230-938_230-937del): Found exclusively in ID 2, with a MAF of 0.92%.

Given their *trans* inheritance, these variants are potential candidate variants to IRIDA in these monoallelic subjects. Further mRNA analysis is required to assess their impact on *TMPRSS6* expression.

The other two VUS were identified in isolated subjects and located in regulatory regions:

- rs78987624 (c.-7001G>A): Found in ID 12, with a MAF of 0.22%, predicted to be in the promoter region (Eukaryotic Promoter Database (EPD)). Given its predicted location, this variant may impact *TMPRSS6* expression.
- rs117575523 (c.*503C>G): Found in ID 3, located in the 3′UTR, with predicted effects on splicing.

Inheritance patterns of these two variants remain underdetermined, limiting conclusions about their role in these individuals. Functional studies, including *TMPRSS6* mRNA expression in patient-derived white cells, could further clarify their impact.

### 3.3. Non-Coding Variants Not Linked to Phenotype Expression

The remaining 27 were unlikely contributors to the phenotype due to high prevalence or co-inheritance with the pathogenic *TMPRSS6* variant, but they provided insights into inheritance patterns.

### 3.4. Cis-Inherited Non-Coding Variants

Family 5, with two symptomatic subjects carrying the exonic c.497delT, also carried two exclusive non-coding variants: rs572610055 (c.363+149G>A) and rs147597581 (c.1223+3059C>T). These variants were also present in other symptomatic subjects with c.497delT (ID 5, 9, 11), suggesting co-inheritance with c.497delT. Both variants were classified as likely benign. Notably, variant c.1223+3059C>T was also observed in ID 31 (exonic variant c.431+5G>T).

Variant rs190894261 (c.-5823C>T) was identified in two unrelated symptomatic subjects (ID 14, ID 31), both carrying the exonic c.431+5G>T. This 5′UTR variant (MAF 0.16%) was classified as likely benign, with no predicted splicing effect. Although segregation studies were not possible in these subjects, its presence in two unrelated subjects with the same exonic variant suggests co-inheritance with c.431+5G>T, making it unlikely to explain the IRIDA phenotype in these subjects.

### 3.5. Prevalent Trans-Inherited Non-Coding Variants

No specific variant was observed in all monoallelic IRIDA subjects. However, a highly prevalent non-coding variant, rs71324841 (c.-7607_-7606dup) in the 5′UTR, was identified in three families (Table 1) and one isolated carrier (ID 3). With a MAF of 39.86% and no predicted splicing effect, this variant was classified as benign (class 1).

Seven *trans-*inherited non-coding variants were exclusive to asymptomatic subjects (Table 4). Although they had low allele frequencies, their presence in asymptomatic subjects indicates they do not contribute to the phenotype. Additionally, seven non-coding variants were observed in all subjects (Supplementary Table S3).

### 3.6. Whole-Exome Sequencing

Whole exome sequencing (WES) was performed on 12 iron-regulating genes in symptomatic monoallelic subjects and their asymptomatic subjects (Supplementary Table S4).

### 3.7. Potential Modifier: CDAN1 Variant in an Asymptomatic Subject

A likely pathogenic heterozygous variant in *Codanin-1* (*CDAN1*, NM_138477.4: c.2059C>T; p.(Arg687Cys)) was identified in an asymptomatic subject from Family 4 (ID 23). *CDAN1* variants are associated with congenital dyserythropoietic anemia type 1 (CDA-1), characterized by macrocytic anemia with low-hepcidin iron overload due to ineffective erythropoiesis [16]. While CDA-1 typically follows recessive inheritance, rare cases have been reported in individuals with a single pathogenic variant [17]. Although unlikely, we cannot exclude that this variant might have modulated the IRIDA phenotype in ID 23 by compensating for the *TMPRSS6*-induced hepcidin dysregulation.

### 3.8. HFE and TFR2 Variants Unlikely to Explain Phenotypic Differences

One symptomatic subject (ID 7, Family 4) carried a VUS in the *TFR2* gene (NM_003227.4; c.1127C>A; p.(Ala376Asp)). Several *HFE* variants were identified in both symptomatic and asymptomatic monoallelic IRIDA subjects. The pathogenic *HFE* variant (NM_000410.4: c.845G>A; p.(Cys282Tyr)) was heterozygous in two symptomatic subjects (ID 6, ID 10) and one asymptomatic subject (ID 26). Another possibly pathogenic *HFE* variant (c.187C>G; p.(His63Asp)) was heterozygous in three symptomatic IRIDA subjects (ID 1, ID 6, ID 9). Notably, ID 1 had the IRIDA phenotype, whereas her mother—who carried the same exonic *TMPRSS6* variant but not the *HFE* p.(His63Asp)- did not.

Since *HFE*- and *TFR2-*related hereditary hemochromatosis (HH) are recessive conditions associated with (low hepcidin induced) iron overload rather than (high hepcidin induced) iron deficiency, these heterozygous variants could theoretically counterbalance the high-hepcidin iron-deficiency phenotype of *TMPRSS6-*related IRIDA [14,18]. The presence of the *TFR2* VUS in a symptomatic IRIDA subject suggests it is unlikely to contribute to the IRIDA phenotype. Similarly, *HFE* variants found in both symptomatic an asymptomatic subjects indicate they do not significantly affect phenotype expression in this monoallelic IRIDA population.

## 4. Discussion

This study aimed to identify potential contributors to phenotypic variation in monoallelic IRIDA, with a focus on non-coding variants in *TMPRSS6*. We identified four candidate non-coding *TMPRSS6* variants that may play a modifying role (Table 3). Two of these (rs80140288 and rs146953827) were inherited *in trans* with a pathogenic exonic *TMPRSS6* variant and predicted to affect splicing, making them the strongest candidates based on both segregation and in silico analyses. The remaining two variants (rs78987624 and rs117575523) were found in isolated symptomatic subjects, and their inheritance could not be established; nevertheless, their predicted regulatory effects suggest they may influence *TMPRSS6* expression. No single pathogenic non-coding variant consistently explained phenotype expression across all symptomatic subjects. Some non-coding variants were co-inherited with exonic variants, but their contribution remains uncertain. Whole-exome sequencing did not provide strong support for polygenic inheritance as a major determinant of IRIDA phenotypic variation.

The central hypothesis was that monoallelic IRIDA results from a ‘two-hit’ mechanism, where an exonic *TMPRSS6* variant together with a *trans-*inherited non-coding variant drives disease expression. We identified four non-coding *TMRPSS6* variants potentially contributing to the IRIDA phenotype in symptomatic subjects. However, no single pathogenic non-coding variant consistently explained phenotype expression across all symptomatic subjects. We did identify the highly prevalent c.-7607_-7606dup, with an allele frequency of 39.86%, but in silico predictions classified this variant as benign. Although genome-wide association studies (GWAS) have linked common *TMPRSS6* polymorphisms to erythropoiesis and iron parameters in the general population, no GWAS has addressed non-coding variants, leaving their role unclear [19,20]. Despite this limitation, highly prevalent variants are unlikely to fully explain disease expression in individual patients. Therefore, we do not consider c.-7607_-7606dup a significant contributor to disease expression in our symptomatic subjects.

One variant of interest, c.*503C>G in the 3′UTR, may contribute to phenotype variability by influencing allele expression, as this region plays a key role in regulating *TMPRSS6* expression [21]. Preferential expression of the pathogenic allele over the wild-type allele could theoretically exacerbate phenotype severity, consistent with prior evidence of allele-specific expression bias in *TMPRSS6* [21]. Although several mechanisms could underlie this differential expression, the regulatory role of the 3′UTR makes variants like c.*503C>G plausible candidates for further investigation.

Beyond variants promoting IRIDA phenotype expression, we identified seven non-coding variants exclusive to asymptomatic subjects. These variants could theoretically exert a protective effect by enhancing *TMPRSS6* expression. However, gain-of-function variants are rare in autosomal recessive disorders such as IRIDA, where loss-of-function variants are typically more prevalent [22,23]. Moreover, to the best of our knowledge, gain-of-function exonic variants are not documented for *TMPRSS6*, making their contribution unlikely.

Interestingly, we observed a mother-daughter pair (ID 8, ID 24) who both exhibited the IRIDA phenotype, although the mother’s symptoms were milder (Supplementary Table S2). This suggests the potential involvement of additional genetic, epigenetic or environmental modifiers. Factors such as age, sex, and environment are known to influence gene expression [6]. Indeed, biallelic IRIDA patients often show more severe phenotype in early childhood, which improves with age, a pattern that may also extend to monoallelic subjects [24].

In exploring polygenic inheritance, WES of a selected panel of iron-regulating genes did not provide strong evidence for this hypothesis in our symptomatic subjects. A previous case study, however, suggested that polygenic inheritance may contribute to the IRIDA phenotype. Specifically, a loss-of-function *TMPRSS6* variant—normally inhibiting the hepcidin-inducing BMP-SMAD pathway- combined with a gain-of-function *ACVR1* variant—which drives hepcidin synthesis independently of BMP 6- led to dysregulated and inappropriately elevated hepcidin production, thereby contributing to disease expression [25]. Although we detected a likely pathogenic *CDAN-1* variant in an asymptomatic subject, its potential role in counteracting the *TMPRSS6* defect via low-hepcidin induced iron overload is considered rare and unlikely [16,17]. Similarly, *HFE* variants were found in both symptomatic and asymptomatic subjects, making them an unlikely explanation for the phenotypic differences in our cohort.

To the best of our knowledge, this is the first full-sequencing study of monoallelic IRIDA subjects, including both symptomatic and asymptomatic individuals. Our cohort includes monoallelic subjects within familial contexts, providing inheritance insights that strengthen the likelihood of certain variants contributing to phenotype expression, particularly when *trans-*inherited. We also clearly defined the IRIDA phenotype using standardized measurements of hepcidin and TSAT, along with calculation of the TSAT/hepcidin ratio, for which age- and sex-specific reference intervals are available [5,10,26]. Given the relatively large sample size and composition of the population, we believe this study is unique and represents a significant step toward understanding the genetic basis of monoallelic IRIDA.

Nevertheless, the data must be interpreted with caution. The identified non-coding variants were predicted by in silico analyses to affect splicing or *TMPRSS6* regulation. Although useful for prioritizing candidate variants, such predictions require functional validation and are particularly uncertain for non-coding regions, where validation frameworks are less well established. Functional follow-up could include quantitative PCR assessment of *TMPRSS6* mRNA levels, particularly for regulatory variants such as the predicted promoter variant identified in our cohort (rs78987624 (c.-7001G>A)). Because *TMPRSS6* is predominantly expressed in hepatocytes, alternative models—including patient-derived induced pluripotent stem cell-derived hepatocyte-like cells or immortalized cell lines with confirmed *TMPRSS6* expression- may serve as alternatives. Reporter assays and minigene constructs could further clarify effects on transcript stability and splicing, and comparative analyses across biallelic IRIDA patients, symptomatic and asymptomatic monoallelic subjects, and related wild-type relatives would provide the most informative context [27,28].

Second, we assumed a two-hit model for monoallelic IRIDA but did not differentiate between classes of pathogenic exonic *TMPRSS6* variants, despite evidence that null variants (e.g., nonsense, frameshift) cause more severe phenotypes than missense variants [29]. Interactions between different pathogenicity classes of exonic and non-coding variants may influence phenotype severity through cumulative effects. Moreover, distinct non-coding variants may affect different domains and functions of matriptase-2, leading to variable impact on protein function.

Third, our WES analysis focused on a predefined panel of iron-regulating genes and excluded genes associated with severe unrelated phenotypes or without a defined OMIM entry for ethical reasons. While this approach minimized incidental findings, it may also have obscured relevant genetic modifiers contributing to monoallelic IRIDA. However, variants in genes such as *ACVR1A* would be expected to produce a dominant phenotype (fibrodysplasia ossificans progressiva) rather than IRIDA, supporting our gene selection strategy.

Finally, allele skewing was observed in a sequenced fragment of intron 6, likely due to PCR bias. To ensure data reliability, we excluded variants from this region (n = 18), which may have resulted in the omission of potentially relevant variants.

## 5. Conclusions

In this exploratory study, we identified four non-coding *TMPRSS6* variants that may act as potential modifiers of the IRIDA phenotype in monoallelic IRIDA subjects, with two *trans-*inherited variants emerging as the strongest candidates based on segregation and predicted effects on splicing. While polygenic inheritance did not appear to be a major contributor in our cohort, we identified a *CDAN1* variant that may have influenced disease expression in one case. Overall, our findings support the possibility that non-coding variation may contribute to phenotypic variability in monoallelic IRIDA, but functional studies will be essential to clarify their biological relevance and to refine our understanding of genotype-phenotype correlations in monoallelic IRIDA subjects.

## Figures and Tables

**Table 1:** Characteristics of *trans*-inherited non-coding variants found within familial context.

Variants Found Exclusively in Symptomatic Subjects				
Non-Coding Variant	rsID	MAF (%)	Exonic Variant	Subject
5′UTR/PR				
c.-7504C>T	rs115067778	3.45	c.1654G>A	20
c.-7293C>T	rs74839029	3.62	c.1654G>A	20
c.-5224G>A	rs59744498	3.37	c.1654G>A	20
c.-3094C>T	rs77087103	2.25	c.1654G>A	20
**Intron 2**				
c.230-938_230-937del	rs146953827	0.92	del prom exon 1–3	2
c.229+945C>T *	rs80140288	0.53	c.497delT	9, 24
**Intron 6**				
c.658+1123_658+1135delinsC **	-	-	c.1714G>A	3
			c.497delT	24
**Intron 8**				
c.1000+517C>T	rs57746379	2.09	c.1654G>A	20
**Intron 10**				
c.1223+1526C>T	rs539552983	0.016	c.497delT	8
**Intron 15**				
c.1868+368G>A	rs148550682	1.46	c.863+1G>T	10
**Variants Found in Both Symptomatic Subjects and Related WT**				
**Non-Coding Variant**	**rsID**	**MAF (%)**	**Exonic Variant**	**Subject**
**5′UTR/PR**				
c.-7607_-7606dup	rs71324841	39.86	c.1336C>T	3
			c.1346G>A	7, 29 (WT)
			c.497delT	8
			c.1654G>A	20
**Intron 2**				
c.230-1353_230-1329dup	rs140612996	18.15	c.1346G>A	7, 29 (WT)
**Intron 3**				
c.363+543C>G	rs13058647	0.0067	c.1346G>A	7, 29 (WT)
**Intron 17**				
c.2278-31G>A	rs41283231	0.16	c.2105G>A	1, 28 (WT)

The left column shows *trans-*inherited non-coding variants selected as potential contributors to the IRIDA phenotype based on familial segregation analysis, independent of pathogenicity classification. The exonic variant refers to the previously identified monoallelic *TMPRSS6* variant. * Variant also identified in an unrelated symptomatic subject carrying the same pathogenic exonic variant (c.497delT; ID 9). ** Variant also found in another symptomatic subject without included relatives, making inheritance pattern determination impossible. Abbreviations: rsID: Reference SNP cluster ID, MAF: minor allele frequency, obtained from the Genome Aggregation Database (gnomAD), del prom exon 1-3: deletion promoter exons 1-3, WT: wild-type, -: not referenced. The bold/color was used to clarify the different locations where the variants were found.

**Table 2:** Characteristics of non-coding variants found within isolated symptomatic subjects.

Variants Exclusively Found in Symptomatic Subjects				
Non-Coding Variant	rsID	MAF (%)	Exonic Variant	Subject
UTR/PR				
c.-7609_-7606dup	rs71324841	13.82	c.431+5G>T	14
c.-7001G>A	rs78987624	0.15	c.1805G>C	12
c.-5823C>T	rs190894261	0.16	c.431+5G>T	14, 31
c.*503C>G	rs117575523	0.057	c.1336C>T	3
**Intron 2**				
c.229+1413A>G	rs228908	7.95	c.1805G>C	12
**Intron 3**				
c.363+149G>A	rs572610055	0.10	c.497delT	5, 8, 9, 11, 24
**Intron 6**				
c.658+1019T>C	rs73160067	1.08	c.1714G>A	4
c.658+1439G>A	-	-	c.1336C>T	3
**Intron 10**				
c.1223+3059C>T	rs147597581	0.089	c.497delT	5, 8, 9, 11, 24
			c.431+5G>T	31
c.1223+3418C>T	rs566491190	0.0032	c.431+5G>T	14
**Intron 13**				
c.1582+762_1582+765dupCATC	rs558830761	1.05	c.497delT	11
**Variants Found in Symptomatic Subjects and Unrelated WT**				
**Non-Coding Variant**	**rsID**	**MAF (%)**	**Exonic Variant**	**Subject**
**5′UTR/PR**				
c.-4578T>G	rs228913	19.11	c.1805G>C	12
			c.431+5G>T	14, 31
			WT	29 (family c.1346G>A)
c.-3586A>G	rs1883276	19.30	c.1805G>C	12
			c.431+5G>T	14, 31
			WT	29 (family c.1346G>A)
c.-1064T>C	rs228909	21.68	c.1805G>C	12
			c.431+5G>T	14, 31
			WT	29 (family c.1346G>A)
**Intron 2**				
c.229+210G>A	rs5995380	20.62	c.1805G>C	12
			c.431+5G>T	14, 31
			WT	29 (family c.1346G>A)
Intron 3				
c.363+409G>C	rs867039861	0.25	c.1714G>A	4
			WT	29 (family c.1346G>A)
**Intron 6**				
c.658+1166_658+1169delTATC	rs746732095	7.72	c.1714G>A	4
			WT	28 (family c.2105G>T)

The left column presents non-coding variants found exclusively in symptomatic subjects or in both symptomatic subjects and unrelated WT subjects. The exonic variant refers to the previously identified pathogenic exonic *TMPRSS6* variant. Abbreviations: rsID: Reference SNP cluster ID, MAF: minor allele frequency, obtained from the Genome Aggregation Database (gnomAD), WT: wild-type, -: not referenced. The bold/color was used to clarify the different locations where the variants were found.

**Table 3:** Summary of the four candidate variants.

Variant	rsID	MAF (%)	Genomic Location	Evidence
c.229+945C>T	rs80140288	0.53	Intron 2	Family segregation
c.230-938_230-937del	rs146953827	0.92	Intron 2	Family segregation
c.-7001G>A	rs78987624	0.15	Promoter region	Isolated subject
c.*503C>G	rs117575523	0.057	3’UTR	Isolated subject

Abbreviations: rsID: Reference SNP cluster ID, MAF: minor allele frequency, obtained from the Genome Aggregation Database (gnomAD); UTR: untranslated region.

**Table 4:** *Trans*-inherited non-coding variants found exclusively in asymptomatic subjects.

Non-Coding Variant	rsID	MAF (%)	Exonic Variant	Subject
**Intron 2**				
c.230-1580C>T	rs543987633	0.064	c.1346A>G	23, 30 (WT)
**Intron 3**				
c.363+750C>T		12.32	c.1654G>A	21
**Intron 4**				
c.431+238G>A	rs75746329	1.12	c.1654G>A	21
**Intron 6**				
c.658+1123A>C	-	-	c.1654G>A	21
**Intron 8**				
c.864-342T>A	-	-	c.1654G>A	21
**Intron 10**				
c.1000+633C>A	-	-	c.1654G>A	21
c.1224-18G>T	rs9610642	0.067	c.2105G>T	15

The left column displays non-coding variants found exclusively in asymptomatic subjects. The exonic variant refers to the previously identified pathogenic exonic *TMPRSS6* variant. Abbereviations: rsID: Reference SNP cluster ID, MAF: minor allele frequency, obtained from the Genome Aggregation Database (gnomAD), WT: wild-type, -: not referenced. The bold/color was used to clarify the different genomic locations of the variants found.

## Data Availability

Data supporting the findings of this study are included within this article and its Supplementary Materials. Additional data may be available from the corresponding authors upon reasonable request.

## Supplementary Materials

The following supporting information can be downloaded at: https://www.mdpi.com/article/10.3390/genes17010074/s1, Supplementary information S1; Table S1: Genes in iron metabolism analyzed by WES. Tables S2: Characteristics of included IRIDA and wild-type subjects [10]. Tables S3: Non-coding variants observed in all subjects. Tables S4: Results WES-analysis.

## References

[1] Finberg K.E. (2009). Iron-Refractory Iron Deficiency Anemia. Semin. Hematol..

[2] Silvestri L., Nai A., Dulja A., Pagani A. (2019). Hepcidin and the BMP-SMAD pathway: An unexpected liaison. Vitam. Horm..

[3] Donker A.E., Schaap C.C., Novotny V.M., Smeets R., Peters T.M., van den Heuvel B.L., Raphael M.F., Rijneveld A.W., Appel I.M., Vlot A.J. (2016). Iron refractory iron deficiency anemia: A heterogeneous disease that is not always iron refractory. Am. J. Hematol..

[4] Nemeth E., Ganz T. (2023). Hepcidin and Iron in Health and Disease. Annu. Rev. Med..

[5] van der Staaij H., Donker A.E., Bakkeren D.L., Salemans J., Mignot-Evers L.A.A., Bongers M.Y., Dieleman J.P., Galesloot T.E., Laarakkers C.M., Klaver S.M. (2022). Transferrin Saturation/Hepcidin Ratio Discriminates TMPRSS6-Related Iron Refractory Iron Deficiency Anemia from Patients with Multi-Causal Iron Deficiency Anemia. Int. J. Mol. Sci..

[6] Hoving V., Korman S.E., Antonopoulos P., Donker A.E., Schols S.E.M., Swinkels D.W. (2022). IRIDA Phenotype in TMPRSS6 Monoallelic-Affected Patients: Toward a Better Understanding of the Pathophysiology. Genes.

[7] Finberg K.E., Heeney M.M., Campagna D.R., Aydınok Y., Pearson H.A., Hartman K.R., Mayo M.M., Samuel S.M., Strouse J.J., Markianos K. (2008). Mutations in TMPRSS6 cause iron-refractory iron deficiency anemia (IRIDA). Nat. Genet..

[8] Jaspers A., Caers J., Le Gac G., Ferec C., Beguin Y., Fillet G. (2012). A novel mutation in the CUB sequence of matriptase-2 (TMPRSS6) is implicated in iron-resistant iron deficiency anaemia (IRIDA). Br. J. Haematol..

[9] Pellegrino R.M., Coutinho M., D’Ascola D., Lopes A.M., Palmieri A., Carnuccio F., Costa M., Zecchina G., Saglio G., Costa E. (2012). Two novel mutations in the tmprss6 gene associated with iron-refractory iron-deficiency anaemia (irida) and partial expression in the heterozygous form. Br. J. Haematol..

[10] Radboudumc Reference Values WCX-TOF MS for Serum Hepcidin-25. https://www.hepcidinanalysis.com/provided-service/reference-values/.

[11] Wallis Y.L., Payne S.J., Mcanulty C., Bodmer D., Sister-mans E., Robertson K., Moore D., Abbs S.J., Deans Z.C., Devereau A. (2013). Practice Guidelines for the Evaluation of Pathogenicity and the Reporting of Sequence Variants in Clinical Molecular Genetics.

[12] Richards S., Aziz N., Bale S., Bick D., Das S., Gastier-Foster J., Grody W.W., Hegde M., Lyon E., Spector E. (2015). Standards and guidelines for the interpretation of sequence variants: A joint consensus recommendation of the American College of Medical Genetics and Genomics and the Association for Molecular Pathology. Genet. Med..

[13] Ellingford J.M., Ahn J.W., Bagnall R.D., Baralle D., Barton S., Campbell C., Downes K., Ellard S., Duff-Farrier C., FitzPatrick D.R. (2022). Recommendations for clinical interpretation of variants found in non-coding regions of the genome. Genome Med..

[14] Girelli D., Busti F., Brissot P., Cabantchik I., Muckenthaler M.U., Porto G. (2022). Hemochromatosis classification: Update and recommendations by the BIOIRON Society. Blood.

[15] Donker A.E., Raymakers R.A., Vlasveld L.T., van Barneveld T., Terink R., Dors N., Brons P.P., Knoers N.V., Swinkels D.W. (2014). Practice guidelines for the diagnosis and management of microcytic anemias due to genetic disorders of iron metabolism or heme synthesis. Blood.

[16] Heimpel H., Schwarz K., Ebnöther M., Goede J.S., Heydrich D., Kamp T., Plaumann L., Rath B., Roessler J., Schildknecht O. (2006). Congenital dyserythropoietic anemia type I (CDA I): Molecular genetics, clinical appearance, and prognosis based on long-term observation. Blood.

[17] Tamary H., Dgany O., Adam M.P., Feldman J., Mirzaa G.M., Pagon R.A., Wallace S.E., Bean L.J.H., Gripp K.W., Amemiya A. Congenital Dyserythropoietic Anemia Type I. GeneReviews^®^.

[18] Powell L.W., Seckington R.C., Deugnier Y. (2016). Haemochromatosis. Lancet.

[19] Benyamin B., Ferreira M.A., Willemsen G., Gordon S., Middelberg R.P., McEvoy B.P., Hottenga J.J., Henders A.K., Campbell M.J., Wallace L. (2009). Common variants in TMPRSS6 are associated with iron status and erythrocyte volume. Nat. Genet..

[20] Beutler E., Van Geet C., te Loo D.M.W.M., Gelbart T., Crain K., Truksa J., Lee P.L. (2010). Polymorphisms and mutations of human TMPRSS6 in iron deficiency anemia. Blood Cells Mol. Dis..

[21] Serre D., Gurd S., Ge B., Sladek R., Sinnett D., Harmsen E., Bibikova M., Chudin E., Barker D.L., Dickinson T. (2008). Differential Allelic Expression in the Human Genome: A Robust Approach To Identify Genetic and Epigenetic Cis-Acting Mechanisms Regulating Gene Expression. PLoS Genet..

[22] Veitia R.A., Caburet S., Birchler J.A. (2018). Mechanisms of Mendelian dominance. Clin. Genet..

[23] Gerasimavicius L., Livesey B.J., Marsh J.A. (2022). Loss-of-function, gain-of-function and dominant-negative mutations have profoundly different effects on protein structure. Nat. Commun..

[24] De Falco L., Totaro F., Nai A., Pagani A., Girelli D., Silvestri L., Piscopo C., Campostrini N., Dufour C., Manjomi F.A. (2010). Novel TMPRSS6 mutations associated with iron-refractory iron deficiency anemia (IRIDA). Hum. Mutat..

[25] Pagani A., Colucci S., Bocciardi R., Bertamino M., Dufour C., Ravazzolo R., Silvestri L., Camaschella C. (2017). A new form of IRIDA due to combined heterozygous mutations of TMPRSS6 and ACVR1A encoding the BMP receptor ALK2. Blood.

[26] Diepeveen L.E., Laarakkers C.M.M., Martos G., Pawlak M.E., Uğuz F.F., Verberne K., van Swelm R.P.L., Klaver S., de Haan A.F.J., Pitts K.R. (2019). Provisional standardization of hepcidin assays: Creating a traceability chain with a primary reference material, candidate reference method and a commutable secondary reference material. Clin. Chem. Lab. Med..

[27] Qian X., Wang J., Wang M., Igelman A.D., Jones K.D., Li Y., Wang K., Goetz K.E., Birch D.G., Yang P. (2021). Identification of Deep-Intronic Splice Mutations in a Large Cohort of Patients With Inherited Retinal Diseases. Front. Genet..

[28] Rodenburg R.J. (2018). The functional genomics laboratory: Functional validation of genetic variants. J. Inherit. Metab. Dis..

[29] De Falco L., Silvestri L., Kannengiesser C., Morán E., Oudin C., Rausa M., Bruno M., Aranda J., Argiles B., Yenicesu I. (2014). Functional and Clinical Impact of Novel Tmprss6 Variants in Iron-Refractory Iron-Deficiency Anemia Patients and Genotype–Phenotype Studies. Hum. Mutat..

