# Supplementary material for: TMPRSS6 Non-Coding Variants in the Expression of Iron Refractory Iron Deficiency Anemia in Monoallelic Subjects
Source: Genes (Basel). 2026 Jan 8;17(1):74. doi: 10.3390/genes17010074 (PMC12841354; doi:10.3390/genes17010074)
Supplement: Supplementary file 1 [file genes-17-00074-s001.zip › genes-4074273-supplementary.pdf]

Supplemental information

Supplemental Information 1: Materials and methods

Amplification and sequencing of TMPRSS6

DNA samples from patients included in the Radboud Biobank were obtained during outpatient clinical visits as part of routine diagnostic care and stored at the Radboud Iron Biobank, where they were stored after sampling according to standardized operating procedures.

Long range-polymerase chain reaction (LR-PCR) was used to ensure a sufficient of *TMPRSS6* DNA for sequencing. The LR-PCR was combined with a touch-down PCR using the TaKaRa LA Taq® DNA Polymerase Hot-Start version kit (TAKARA BIO) to enhance specificity.
The LR-PCR program was as follows:
1. Initial denaturation at 95°C for 2 minutes
2. Touch down phase (5 cycles):
- 95°C for 20 seconds
- 68°C for 30 seconds (decreasing by 1°C per cycle)
- 68°C for 11 minutes
3. Amplification phase (30 cycles)
- 95°C for 20 seconds
- 64°C for 30 seconds
- 68°C for 11 minutes
4. Final elongation:
- 68°C for 15 minutes
5. Storage:
- Samples were kept at 10°C post-amplification, and eventually stored at -20°C until further use.

Following amplification, the PCR products were analyzed on an 1% agarose gel prepared with a 150 ml tray with 1.5 grams of agarose dissolved in 150 ml of 1x Tris-borate-EDTA (TBE) buffer. To visualize the DNA bands, 60 µl Nancy-520 was added to the liquid 1% agarose. For gel loading, 1.0 µl of the PCR product was mixed in a 1:1 ratio with 2x bromophenol blue (BPB) loading dye. This mixture, along with 5.0 µl of 1 Kb Plus DNA Ladder (Thermo Fisher), was loaded onto the gel. The gel ran at 100-120 V for 60-90 minutes, and the results were analyzed using a Universal Hood II GelDoc System (Bio-Rad).

PacBio SMRT Sequencing of TMPRSS6

All samples were pooled, resuspended, plated and sent for Single Molecule, Real-Time (SMRT) PacBio sequencing at the Genetics Department of Radboudumc.
DNA quantification and pooling:
- The dsDNA concentration was measured using the Invitrogen™ Qubit™ 4 Fluorometer (Thermo Fisher).
- For sequencing, 500 ng of dsDNA in 50 µl VERSOL was used
- To prevent pipetting errors when handling low-volume LR-PCR products (<1 µL), 2500 ng from each sample was pooled in 250 µL VERSOL before final pooling.
- Since *TMPRSS6* was amplified into six different DNA fragments of varying sizes, a correction was applied to ensure an equal amount of dsDNA molecules per fragment before

pooling. Using the measured dsDNA concentrations paired with the pre-determined ratios, the required amount of pooled dsDNA was calculated. Subsequently, 50 µl was sent for SMRT PacBio sequencing.

Supplemental Table S1. Genes in iron metabolism analyzed by WES

Gene	MIM number	Associated disorder (including phenotype MIM number)
**CDAN1**	607465	Congenital dyserythropoietic anemia type 1A (224120)
**FTH1**	134770	Hemochromatosis type 5 (615517)
**HAMP**	606464	Hemochromatosis 2B (613313)
**HFE**	613609	Hemochromatosis (235200)
**HJV**	608374	Hemochromatosis type 2A (602390)
**HSCB**	608142	Sideroblastische anemie type 5 (619523)
**KIF23**	600599	Bloodgroup - Lutheran inhibitor (111150)<br>Congenital dyserythropoietic anemia type IV (613673)
**SLC11A2**	600523	Hypochromic, microcytic anemia with iron loading type 1 (206100)
**SLC25A38**	610819	Sideroblastic anemia type 2, pyridoxin refractory (205950)
**SLC40A1**	604653	Hemochromatosis type 4 (606069)
**TF**	190000	Atransferrinemia (209300)
**TFR2**	604720	Hemochromatosis type 3 (604250)

The left column lists genes selected for WES-analysis. *MIM: Mendelian Inheritance in Man. CDAN1: codanin-1; FTH: ferritin heavy chain 1; HAMP: hepcidin antimicrobial peptide; HFE: homeostatic iron regulator; HJV: hemojuvelin; HSCB: heat shock cognate B; KIF23: kinesin family member 23; SLC11A2: solute carrier family 11; SLC25A38: solute carrier family 25, member 38; SLC40A1: solute carrier family 40, member 1; TF: transferrin; TFR2: transferrin receptor 2.*

Supplemental Table S2. Characteristics of included IRIDA and wild-type subjects.

	Relation	Subject	Age (yrs)	Hb (g/dL)	MCV (fl)	Ferritin (ug/L)	TSAT (%)	TSAT/hepc^	WES result
**Family 1**									
**1**	Proband	SS	29	9.7	68	22	4.0	0.52	HFE: class 4
**15**	Mother	AS	60	14.5	95	56	21.9	12.9	
**28**	Father	WT	62	16.1	85	98	38.2	22.5	
**Family 2**									
**2**	Proband	SS	36	5.5	76	238*	7.0*	1.3*	
**17**	Father	AS	71	10.6	86	151	14.4	11.1	
**18**	Aunt (maternal)	AS	66	13.3	90	128	30.7	n.a.	
**Family 3**									
**20**	Proband	SS	43	7.9	62	3.0	2.8	0.5	
**21**	Sister	AS	38	11.9	92	68	18.3	7.3	
**Family 4**									
**7**	Proband	SS	3	9.8	60	50	4	0.8	TFR2: VUS
**23**	Mother	AS	34	13.7	89	55	28	n.a.	CDAN-1: class 4
**29**	Father	WT	35	13.8	85	226	31	n.a.	
**30**	Sister	WT	7	n.a.	n.a.	n.a.	n.a.	n.a.	
**Family 5**									
**8**	Proband	SS	32	10.0	76	32	4.0	0.3	
**24**	Mother	SS	58	12.6*	86	299	13	0.6	
**Family 6**									
**10**	Proband	SS	41	8.9	71	34	6.0	1.0.	HFE: class 5
**25**	Sister	AS	54	12.9	77	88	15	3.1	
**26**	Mother	AS	83	10.0	84	502**	18.8	0.8	HFE: class 5
**28**	Brother	WT	50	15.0	79	217	32.1	6.0	

Overview of laboratory results at time of presentation with anemia. The right column represents results from WES analysis of additional iron-regulating genes.
*After IV iron supplementation, **CRP level increased; 45mg/L.
Hb: hemoglobin, MCV: mean corpuscular volume, TSAT: transferrin saturation, TSAT/hepc: transferrin saturation/hepcidin ratio. SS: symptomatic subject, AS: asymptomatic symptomatic, WT: subject with wild-type genotype. HFE: High Fe^{2+} *gene, TFR2: transferrin receptor 2, CDAN-1: codanin-1*
^ Reference values are available at hepcidinanalysis.com [1]

Supplemental Table S3. Non-coding variants observed in all subjects.

Variant	rs	Location	MAF	ACMG (Alamut)
**c.363+521A>T**	rs228900	Intron 3	66.33%	BA1
**c.863+23A>G**	rs2235326	Intron 7	59.59%	BA1/BP6
**c.1223+2081T>C**	rs1005478	Intron 10	66.59%	BA1
**c.1223+2603G>A**	rs2543523	Intron 10	61.89%	BA1
**c.1223+4142T>C**	rs2743819	Intron 10	76.96%	BA1
**c.1223+4192G>A**	rs1558955	Intron 10	55.76%	BA1
**c.1582+913G>C**	rs855790	Intron 13	97.08%	BA1

The left column displays identified non-coding variants. *Rs-number: reference single nucleotide polymorphism (rs)ID in dbSNP. MAF: minor allele frequency, obtained from the Genome Aggregation Database (gnomAD). ACMG: classification by Alamut software.*

Supplemental Table S4. Results WES-analysis

ID		1	15	2	17	18	7	23	10	25	26	20	21	8	24	3	4	5	6	9	11	12	14	31
**CDAN1**																								
variant	hetero/homozygous	class	class	class	class	class	class	class	class	class	class	class	class	class	class	class	class	class	class	class	class	class	class	class
NM_138477.4(CDAN1):c.3194G>A, p.(Arg1065Gln	heterozygous	2	-	-	-	-	-	-	-	-	-	-	-	-	-	-	-		-	-	-	-	-	-
NM_138477.4(CDAN1):c.3474A>C, p.(Leu1158=)	heterozygous	-	-	-	-	-	-	1	-	-	-	-	-	1	1	-	-	1	-	-	-	-	1	1
NM_138477.4(CDAN1):c.2671C>T, p.(Arg891Cys)	heterozygous	-	-	-	-	-	-	-	-	-	-	1	-	1	1	-	-	1	-	-	-	1	1	1
NM_138477.4(CDAN1):c.2408-3C>T, p.?	heterozygous	-	-	-	-	-	-	-	-	-	-	1	-	1	1	-	-	1	-	-	-	1	1	1
NM_138477.4(CDAN1):c.1787A>G, p.(Gln596Arg)	heterozygous	-	-	-	-	-	-	-	-	-	-		-	1	1	-	-	1	-	-	-	-	1	1
NM_138477.4(CDAN1):c.477C>T, p.(Pro159=)	heterozygous	-	-	-	-	-	-	1	-	-	-	1	-	1	1	-	-	1	-	-	-	1	1	1
NM_138477.4(CDAN1):c.320A>T, p.(Gln107Leu)	heterozygous	-	-	-	-	-	-	1	-	-	-		-	1	1	-	-	1	-	-	-	-	1	1
NM_138477.4(CDAN1):c.2352+8C>T, p.?	heterozygous	-	-	-	-	-	-	-	-	-	-	1	1	1	1	-	-	-	-	-	-	-	-	-
NM_138477.4(CDAN1):c.816C>A p.(Thr272=)	heterozygous	-	-	-	-	-	-	-	-	-	-	1	-	-	-	-	-	-	-	-	-	-	-	-
NM_138477.4(CDAN1):c.3474A>C, p.(Leu1158=)	homozygous	-	-	-	-	-	-	-	-	-	-	1	1	-	-	-	-	-	-	-	-	1	-	-
NM_138477.4(CDAN1):c.320A>T, p.(Gln107Leu)	homozygous	-	-	-	-	-	-	-	-	-	-	1	1	-	-	-	-	-	-	-	-	1	-	-
NM_138477.4(CDAN1):c.1787A>G, p.(Gln596Arg)	homozygous	-	-	-	-	-	-	-	-	-	-	1	1	-	-	-	-	-	-	-	-	-	-	-
NM_138477.4(CDAN1):c.2671C>T, p.(Arg891Cys)	homozygous	-	-	-	-	-	-	-	-	-	-	-	1	-	-	-	-	-	-	-	-	-	-	-
NM_138477.4(CDAN1):c.2408-3C>T, p.?	homozygous	-	-	-	-	-	-	-	-	-	-	-	1	-	-	-	-	-	-	-	-	-	-	-
NM_138477.4(CDAN1):c.477C>T, p.(Pro159=)	homozygous	-	-	-	-	-	-	-	-	-	-	-	1	-	-	-	-	-	-	-	-	-	-	-
NM_138477.4(CDAN1):c.1969G>A p.(Gly657Ser)	heterozygous	-	-	-	-	-	-	-	-	-	-	-	1	-	-	-	-	-	-	-	-	-	-	-
NM_138477.4(CDAN1):c.2059C>T p.(Arg687Cys)	heterozygous	-	-	-	-	-	-	4	-	-	-	-	-	-	-	-	-	-	-	-	-	-	-	-
**FTH1**																								
variant	hetero/homozygous	class	class	class	class	class	class	class	class	class	class	class	class	class	class	class	class	class	class	class	class	class	class	class
NM_002032.3(FTH1):c.456C>T p.(His152=)		-	-	-	-	-	-	-	-	-	-	2	-	-	-	-	-	-	-	-	-	-	-	-
							-																	
**HAMP**																								
variant	hetero/homozygous	class	class	class	class	class	class	class	class	class	class	class	class	class	class	class	class	class	class	class	class	class	class	class
No variants		-	-	-	-	-	-	-	-	-	-	-	-	-	-	-	-	-	-	-		-	-	-
																					-			
**HFE**																								
variant	hetero/homozygous	class	class	class	class	class	class	class	class	class	class	class	class	class	class	class	class	class	class	class	class	class	class	class
NM_000410.4: c.187C>G, p.(His63Asp)	heterozygous	4	-	-	-	-	-	-	-	-	-	-	-	-	-	-	-	-	4	4	-	-	-	-
NM_000410.4:c.340+4T>C p.?)	heterozygous	1	-	-	-	-	-	1	1	1	-		1	-	-	1	-	1	1	1	-	-	1	
NM_000410.4(HFE):c.845G>A, p.(Cys282Tyr)	heterozygous	-	-	-	-	-	-	-	5	-	5	-	-	-	-	-	-	-	5	-	-	-	-	-
NM_000410.4:c.340+4T>C p.?)	homozygous	-	-	-	-	-	-	-	-	-	-	1	-	-	-	-	-	-	-	-	-	1	-	-
NM_000410.4(HFE):c.193A>T p.(Ser65Cys)	heterozygous	-	-	-	-	-	-	-	-	-	-	-	-	-	-	-	-	-	-	-	-	-	-	-
NM_000410.4(HFE):c.617-48C>G p.?	heterozygous	-	-	-	-	-	-	-	-	-	-	-	1	-	-	-	-	-	-	-	-	-	-	-
**HJV**																								
variant	hetero/homozygous	class	class	class	class	class	class	class	class	class	class	class	class	class	class	class	class	class	class	class	class	class	class	class
NM_000410.4(HFE):c.340+4T>C p.?	homozygous	-	-	-	-	-	-	-	-	-	-	1	-	-	-	-	-	-	-	-	-	-	-	-
							-																	
**HSCB**																								
variant	hetero/homozygous	class	class	class	class	class	class	class	class	class	class	class	class	class	class	class	class	class	class	class	class	class	class	class
No variants		-	-	-	-	-	-	-	-	-	-	-	-	-	-	-	-	-	-	-	-	-	-	-

ID		1	15	2	17	18	7	23	10	25	26	20	21	8	24	3	4	5	6	9	11	12	14	31
KIF23																								
variant	hetero/homozygous	class	class	class	class	class	class	class	class	class	class	class	class	class	class	class	class	class	class	class	class	class	class	class
NM_138555.4c.1485A>G, p.(Pro495=)	homozygous	1	-	-	-	-	-	-	-	-	-	-	-	-	-	-	-	-	-	-	-	-	-	-
NM_138555.4c.2019C>T, p.(Thr673=)	homozygous	1	-	-	-	-	-	-	-	-	-	-	-	-	-	-	-	-	-	-	-	-	-	-
NM_138555.4(KIF23):c.152C>T, p.(Thr51Ile)	heterozygous	-		1	1		-		-			-		-		-	-	1	-	-	-	-	-	-
NM_138555.4(KIF23):c.1326A>C, p.(Val442=)	heterozygous	-		1	-		-		-			-		-		-	-	-	-	-	-	-	-	-
NM_138555.4(KIF23):c.1443A>G, p.(Pro481=)	homozygous	-	1	1		1	-	1	1	1	1	1	1	1	1	1	1	1	1	1	1	-	1	1
NM_138555.4(KIF23):c.1977C>T, p.(Thr659=)	homozygous	-	1	1	1	1	-	1	1	1	1	1	1	1	1	1	1	1	1	1	1	-	1	1
NM_138555.4(KIF23):c.1443A>G, p.(Pro481=)	heterozygous	-	-	-	1	-	-	-	-	-	-	-	-	-	-	-	-	-	-	-	-	1	-	-
NM_138555.4(KIF23):c.1977C>T, p.(Thr659=)	heterozygous	-	-	-	-	-	-	-	-	-	-	-	-	-	-	-	-	-	-	-	-	1	-	-
NM_138555.4(KIF23):c.108C>A, p.(Gly36=)	heterozygous	-	-	-	-	-	-	-	-	-	-	-	-	-	-	-	-	-	-	-	-	1	-	-
							-																	
SLC11A2							-																	
variant	hetero/homozygous	class	class	class	class	class	class	class	class	class	class	class	class	class	class	class	class	class	class	class	class	class	class	class
NM_000617.3:c.1254T>C, p.(Ile418=)	heterozygous	1	1	1	1	-	-	-	-	-	-	1	-	1	1	-	-	1	-	1	-	1	1	1
NM_000617.3:c.35-498A>C p.(?)	homozygous	1	1	1	1	1	-	-	1	-	1	1	1	1	1	1	1	1	1	1	-	1	1	1
NM_000617.3:c.35-498A>C p.(?)	heterozygous	-	-	-	-	-	-	1	-	1	-	1	-	-	-	-	-	-	-	-	1	-	-	-
SLC25A38																								
variant	hetero/homozygous	class	class	class	class	class	class	class	class	class	class	class	class	class	class	class	class	class	class	class	class	class	class	class
NM_017875.4c.165G>A, p.(Leu55=)	heterozygous	1	-	1	1	-	-	1	1	-	1	-	-	-	-	-	1	-	1	1	1	1	-	-
NM_017875.4(SLC25A38):c.165G>A, p.(Leu55=)	homozygous	-	-	-	-	1	-		-	-		-		-	-	1	-	-	-	-	-	-	-	1
NM_017875.4(SLC25A38):c.239C>G,p.(Thr80Arg)	heterozygous	-	-	-	-	-	-	-	-	-	-	-	-	1	-	-	-	1	-	-	-	-	-	-
							-																	
SLC40A1							-																	
variant	hetero/homozygous	class	class	class	class	class	class	class	class	class	class	class	class	class	class	class	class	class	class	class	class	class	class	class
NM_014585.6(SLC40A1c.663T>C, p.(Val221=)	homozygous	1	1	1	-	-	1	-	-	-	-	-	-	1	-	1	1	-	1	1		1	-	-
NM_014585.6(SLC40A1c.663T>C, p.(Val221=)	heterozygous	-	-	-	1	1	-	1	1	1	1	1	1	-	1	-	-	-	-	-	1	-	1	1
NM_014585.6(SLC40A1):c.327C>T p.(Ile109=)		-	-	-	-	-	-	-	-	-	-	1	-	-	-	-	-	-	-	-	-	-	-	-
TF																								
variant	hetero/homozygous	class	class	class	class	class	class	class	class	class	class	class	class	class	class	class	class	class	class	class	class	class	class	class
NM_001063.4(TF):c.1765C>T p.(Pro589Ser)	heterozygous	1		-	-	-	-		-			-		1		-	1	-		-	-	-	-	-
NM_001063.4(TF):c.1342A>G p.(Ile448Val)	homozygous	1	1	1	1	1	1	1	1	1	1	1	1	1	1	1	1	1	1	1	1	1	1	1
NM_001063.4(TF):c.624G>A p.(Ser208=)	heterozygous	1	1	-	-	-	1	1	1	1	1	-	-	-	-	-	-	1	-	1	1	1	1	-
NM_001063.4(TF):c.1572G>C, p.(Leu524=)	homozygous	-	-	1	1	-	-	-	-	-	-	-	-	-		-	-	-	-	-	-	-	-	-
NM_001063.4(TF):c.739C>T, p.(Leu247=)	heterozygous	-	-	-		1	-		-	1	-		1	-	1	1	-	-	-	-	1	-	1	1
NM_001063.4(TF):c.829G>A, p.(Gly277Ser)	heterozygous	-	-	-	-	1	-		-	-	-	-	1	-	-	1	-	-	-	-	1	-	-	1
NM_001063.4(TF):c.333G>A, p.(Gln111=)	heterozygous	-	-	-	-		-		-	-	-	-		-	-	-	2	-	-	-	-	-	-	-
NM_001063.4(TF):c.1572G>C, p.(Leu524=)	heterozygous	-	-	-	-	1	1	1	-	-	-	1		-	-	-	1	1	-	-	-	-	-	-
NM_001063.4(TF):c.1765C>T p.(Pro589Ser)	homozygous	-	-	-	-	-	-	-	-	-	-	-	-	-	-	-	-	-	1	-	-	-	-	-
NM_001063.4(TF):c.1572G>C, p.(Leu524=)	heterozygous	-	-	-	-	-	-	-	-	-	-	-	-	-	-	-	-	-	-	-	-	-	-	1
NM_001063.4(TF):c.119G>A p.(Ser40Asn)	heterozygous	-	-	-	-	-	-	-	-	2	-	-	-	-	-	-	-	-	-	-	-	-	-	-

ID		1	15	2	17	18	7	23	10	25	26	20	21	8	24	3	4	5	6	9	11	12	14	31
TFR2																								
variant	hetero/homozygous	class	class	class	class	class	class	class	class	class	class	class	class	class	class	class	class	class	class	class	class	class	class	class
NM_003227.4(TFR2);c.1851C>T, p.(Ala617=)	heterozygous	1	-	-	-	1	-	1		-	-	-	-	-	-	1	1	-	-	1	-	-	1	1
NM_003227.4(TFR2);c.1851C>T, p.(Ala617=)	homozygous	-	-	-	-	-	-	-	-	-	-	-	-	-	-	-	-	-	1	-	-	-	-	-
NM_003227.4(TFR2):c.1127C>A p.(Ala376Asp)	heterozygous	-	-	-	-	-	3	-	-	-	-	-	-	-	-	-	-	-	-	-	-	-	-	-
NM_003227.4(TFR2):c.1449C>T p.(Ser483=)	heterozygous	-	-	-	-	-	-	2	-	-	-	2	2	-	-	-	-	-	-	-	-	-	-	-

The left column displays the selected genes for Whole Exome Sequencing (WES), with identified variants listed below each corresponding gene. ID numbers corresponds to IRIDA subjects as mentioned in the manuscript and are shown in the first row. ID numbers are grouped by family, with red-marked IDs indicating symptomatic subjects, green-marked indicating asymptomatic subjects. WT-subjects are not included in this analysis. Variants classified as class 3 or higher are marked in red.

Reference

1. Hepcidinanalysis.com. Reference values WCX-TOF MS for serum Hepcidin-25.: Internet; [Available from: https://www.hepcidinanalysis.com/provided-service/reference-values/.
